# Evaluation of Emerging Technologies to Aid in the Detection and Diagnosis of Acute Extremity Compartment Syndrome

**DOI:** 10.3390/diagnostics15202607

**Published:** 2025-10-16

**Authors:** Catharina Gaeth, Daniel J. Cognetti, Stefanie M. Shiels, Kinton Armmer, Amber M. Powers, Robert V. Hainline, Thomas J. Walters, Robert J. Moritz

**Affiliations:** Combat Wound Care, United States Army Institute of Surgical Research, 3698 Chambers Pass, Bldg. 3611, Fort Sam Houston, TX 78234-6315, USA; catharina.c.gaeth.fm@health.mil (C.G.); daniel.j.cognetti.mil@health.mil (D.J.C.); stefanie.m.shiels.civ@health.mil (S.M.S.); kinton.l.armmer.civ@health.mil (K.A.); amber.m.powers2.ctr@health.mil (A.M.P.); robert.v.hainline.mil@health.mil (R.V.H.); thomas.j.walters22.ctr@health.mil (T.J.W.)

**Keywords:** acute extremity compartment syndrome, pre-clinical research, animal models, COTS, diagnostics, NIRS

## Abstract

**Background/Objectives**: The diagnosis of acute compartment syndrome (ACS) of the extremities is typically based on subjective clinical signs and symptoms, highlighting the need for user-friendly diagnostic tools to improve accuracy and reliability. This study evaluates the performance of two commercial devices, the MY01^®^ continuous pressure monitoring system and the Moxy Monitor near-infrared spectroscopy-based system, against a reference standard of continuous intracompartmental pressure (ICP) monitoring in a preclinical ACS model. **Methods**: ACS was induced in the anterior compartment of the distal hind limb in eight Yorkshire pigs using a balloon displacement model. ICP was incrementally elevated and maintained for four hours at >30 mmHg above mean arterial pressure. This was followed by balloon deflation and reperfusion. Final assessments were performed at 24 h post-injury. ICP measurements from the MY01^®^ and muscle oxygen saturation (SmO_2_) data from the Moxy Monitor were compared to reference ICP measurements. Histologic analysis of muscle tissue was performed to assess the severity of necrosis. **Results**: The MY01^®^ provided accurate ICP measurements, with a mean bias of 2.21 ± 18.77 mmHg during pre-ischemia, 4.86 ± 10.43 mmHg during reperfusion, and 4.69 ± 3.28 mmHg 24 h post-injury, compared to reference probes. Correlation at 24 h post-injury was (r = 0.86, R^2^ = 0.73, *p* < 0.0001). In contrast, the Moxy Monitor failed to detect significant differences in SmO_2_ between injured and control limbs at 24 h post-injury, despite pronounced ICP differences. Our volumetric displacement ACS model demonstrated its efficacy as a testing platform by allowing for controlled, incremental elevation in ICP and sustaining elevated ICP levels after 24 h. Histologic evaluation confirmed extensive muscle damage, including edema and necrosis. **Conclusions**: The MY01^®^ provides accurate, continuous ICP monitoring, supporting its clinical utility in ACS diagnosis. However, the use of near-infrared spectroscopy-based systems such as the Moxy Monitor for ACS diagnosis and management should continue to be critically scrutinized.

## 1. Introduction

Acute extremity compartment syndrome (ACS) is a severe, potentially limb-threatening condition resulting from various causes, such as long bone fractures, vascular injuries, edema, burns, prolonged limb compression, or crush injuries [1,2]. This can lead to elevated intracompartmental pressure (ICP) which compromises tissue perfusion and oxygen delivery [3,4]. Without timely intervention, ACS can lead to irreversible tissue damage and permanent loss of limb function. The estimated incidence of ACS is 7.3 cases per 100,000 in men and 0.7 per 100,000 in women, with most cases occurring after trauma [5].

The pathophysiology of ACS begins with trauma-induced swelling, hemorrhage, or reperfusion within the confined space of a muscular compartment [6]. The noncompliant fascia surrounding the muscles cannot expand to accommodate the increased volume, resulting in elevated ICP. As ICP rises, perfusion pressure decreases, resulting in tissue hypoxia and a shift from aerobic to anaerobic metabolism. This triggers a cascade of ischemic changes, inflammation, and neurovascular compromise, with prolonged elevations of ICP exacerbating these processes, leading to progressive tissue damage and worsening outcomes [7,8,9].

Considering the potential consequences of delayed or missed ACS diagnosis, particularly in unconscious or uncooperative patients, there is a pressing need for improved diagnostic strategies, given traditional clinical signs and symptoms are highly subjective and often unreliable and can be quickly overlooked [10,11,12]. In this context, indicators such as increased ICP, central to the pathophysiology of ACS, and downstream metrics of tissue perfusion, such as muscle oxygenation, are increasingly recognized as potential adjuncts for diagnosing ACS and monitoring its progression [13,14]. In addition, continuous monitoring provided by other trained medical personnel may be particularly useful in reducing workload of the clinician. However, despite significant research, their adoption in clinical practice remains limited. This is partly due to diagnostic uncertainty and poor interobserver reliability, particularly with early commercial ICP devices and those which utilize single time-point assessments [15]. In contrast, continuous ICP monitoring has shown improved accuracy and diagnostic utility, yet its more involved setup and limited portability have hindered widespread adoption [12]. Similarly, near-infrared spectroscopy (NIRS) for muscle oxygenation has shown promise as a non-invasive alternative, but existing devices remain limited by size, cost, and diagnostic reliability [16,17,18,19,20,21].

Therefore, the objective of this study was to evaluate the diagnostic performance of two commercial-off-the-shelf devices, the MY01^®^ continuous compartment pressure monitor (NXTSens Inc., Montreal, QC, Canada) and the Muscle Oxygen (Moxy) Monitor (Fortiori Design, LLC, Hutchinson, MN, USA). Device selection in this study prioritized availability and portability. In this study, we refine an existing intracompartmental balloon model of ACS in swine showing a novel persistence of ICP elevation with physiologic features that more closely align to a clinical scenario. Using this model, we compared these devices against a continuous ICP reference standard to assess their accuracy, reliability, and potential clinical utility.

## 2. Materials and Methods

### 2.1. Animals

Eight female Yorkshire swine (Midwest Research Swine, Gibbon, MN, USA) were acclimated for a minimum of 72 h following transport and provided commercial feed and water ad libitum. The animals were 47.44 ± 2.4 kg and fasted overnight at the time of procedures. Research was conducted in compliance with the Animal Welfare Act, the implementation of Animal Welfare regulations, and the principles of the Guide for the Care and Use of Laboratory Animals. The Institutional Animal Care and Use Committee approved all research conducted in this study (A-23-013, approved on 15 March 2023). The facility where this research was conducted is fully accredited by the AAALAC International. Experimental details are reported following the ARRIVE (Animal Research: Reporting of In Vivo Experiments) guidelines.

### 2.2. Commercially Available Devices

Two commercially available devices were selected for evaluation. Device selection in this study prioritized availability and portability. The MY01^®^ is a minimally invasive device for continuous real-time measurement of ICP [22]. The device is equipped with a Microelectromechanical System (MEMS) sensor that is percutaneously inserted into the muscle compartment with a 17-gauge needle and can monitor pressures ranging from −99.9 to 99.9 mmHg [22,23]. The Moxy Monitor, designed for use in sports and training, measures muscle oxygen saturation (SmO_2_) by analyzing the balance between oxygen supply and consumption in muscle tissue [24]. This device uses a combination of light-emitting diodes (LEDs), at wavelengths from 630 to 850 nm and two photodetectors to assess the ratio of oxygenated hemoglobin to total hemoglobin concentration, providing continuous feedback of muscle oxygenation [24].

### 2.3. Animal Model Timeline and General Procedures

In this study, we refine an existing intracompartmental balloon model of ACS in swine showing a novel persistence of ICP elevation with physiologic features that more closely align to a clinical scenario. Using this model, we compared these devices against a continuous ICP reference standard to assess their accuracy, reliability, and potential clinical utility.

For each procedure day, anesthesia was induced with tiletamine–zolazepam (Telazol, 4 mg/kg, IM). Animals were intubated and anesthesia was maintained with isoflurane in oxygen until all procedures were complete, at which time they were transferred to their cage for recovery. Twenty-four hours prior to the induction of the ischemic injury, the animals received pre-emptive analgesia (Buprenorphine SR/ER, 0.24 mg/kg) and the carotid artery was cannulated via standard cut-down procedure under general anesthesia using aseptic technique. Additional analgesia was administered for breakthrough pain, depending on daily pain assessments carried out by experienced personnel.

**Figure 1:**
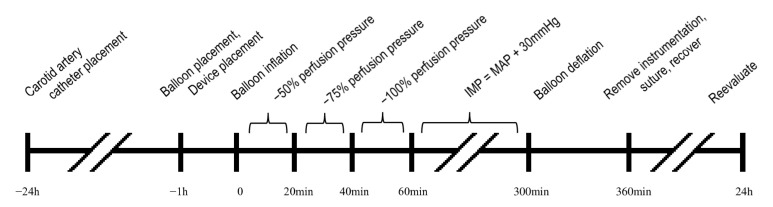
Experimental timeline and methodology. IMP, intramuscular pressure; MAP, mean arterial pressure.

ACS was induced using the intracompartmental balloon model adapted from Honjol et al. [25]. On the day of ACS induction, the animals were placed in a supine position under general anesthesia. A 6.4 mm trocar was inserted in the right hind limb from the medial distal aspect of the anterior compartment towards the lateral proximal aspect, following the path of least resistance between the anterior tibial surface and the anterior muscle compartment. A sterile plastic surgical tubing was passed over the trocar, and the trocar was removed, leaving the surgical tubing in place. An 18 × 40 mm balloon angiocatheter (C.R. Bard Inc., Murray Hill, NJ, USA) was advanced through the surgical tubing and the surgical tube was removed with the balloon angiocatheter remaining in the limb. The contralateral control limb was not subjected to any procedure. The animals were placed in a right-side lateral recumbent position for the remainder of the ischemic injury procedure. Mikro-tip^®^ catheter transducers (Millar, Houston, TX, USA) were inserted percutaneously while connected to a data acquisition system after calibration in accordance with the manufacturer’s instructions. The probes were guided through the lumen of a 16-gauge IV catheter (Terumo, Somerset, NJ, USA) at angles from 30° to 45° to the skin in a proximal direction and advanced to the mid belly of the peroneus tertius muscle in accordance with the manufacturer’s instructions. Two probes were inserted into each limb. The MY01^®^ probe was placed only in the injured limb, using the same approach as the control probes. Reproducibility of pressure changes was checked with passive dorsiflexion of the ankle and probes were secured with non-circumferential adhesive tape. Moxy Monitors were placed on the skin, overlaying anterior compartments of injured and contralateral hind limbs, and secured with opaque tape. ICP values from Mikro-Tip^®^ catheter transducers were averaged and used as an ICP reference for angiocatheter balloon inflation volumes, and differences between duplicate reference probes served as an indicator of variability. The carotid artery cannula was used to monitor and record hemodynamic parameters throughout the experiment (Supplementary Material S1: Picture: Experimental Set Up). Following the probe placement, the balloon was gradually inflated with saline to elevate ICP. The ICP was elevated to 50%, 75%, and 100% of the diastolic pressure (ΔP) over a period of 60 min at 20 min intervals (Figure 1). After one hour of stepwise ICP elevation (pre-ischemia phase), the balloon angiocatheter was further inflated until the ICP exceeded the mean arterial pressure (MAP) by >30 mmHg. This elevated pressure was maintained for four hours to produce prolonged ischemia.

After five hours of elevated ICP, the balloon angiocatheter was fully deflated, and monitoring continued for the first hour of reperfusion. Following this period, the balloon angiocatheter and monitoring probes were removed. The balloon insertion sites were closed, gauze and Tegaderm^TM^ dressing were applied, and the swine were allowed to recover.

Approximately 24 h after the initial onset of injury, the animals were re-anesthetized and placed in a lateral recumbent position. Mikro-Tip^®^ catheter transducers, MY01^®^ devices, and Moxy Monitors were placed as previously described and hemodynamic parameters, ICP and SmO_2_, were recorded over a two-hour period to assess injury progression. Twenty minutes before the end of the two-hour monitoring period, Moxy Monitors were removed and a fasciotomy was performed. An incision was made through the skin and fascia of the injured anterior compartment from the proximal to the distal aspect of the limb (approximately 10–12 cm) to achieve decompression. The animals were then humanely euthanized, and the tissue was collected for histology.

### 2.4. Histology

Peroneus tertius muscle cross sections were fixed in 10% buffered formalin, paraffin embedded, sliced, and stained with hematoxylin/eosin. Samples were blinded to procedural limb, then evaluated by an independent histopathologist using a predetermined histological grading scale with categories standardized at the institution. Histologic grading included changes in skeletal muscle morphology, myocyte appearance, intermyocyte hemorrhage and edema, and inflammation as indicated by the presence of neutrophils, histiocytes, and lymphocytes (Supplementary Material S2: Histologic Evaluation).

### 2.5. Statistical Analysis

Statistical testing was carried out using GraphPad Prism 10.4 (GraphPad Software LLC., La Jolla, CA, USA). Continuous outcomes were tested for normality using Q–Q plots and the D’Agostino–Pearson test. Normally distributed data were expressed as mean and standard deviation (SD) (SEM or 97.5% confidence intervals if applicable) and were tested for mean differences between experimental groups. Group sizes were determined using the SD of ICP compared from previous studies that employed the MY01^®^ device [25] (±5.0 mmHg) and a pilot study that employed Mikro-Tip^®^ catheter transducers (±5.467 mmHg). A sample size of *n* = 8 with an alpha of 0.01 provided 83% power to observe statistical differences using a two-sample *t*-test. Statistical bias of ICP measured by MY01^®^ devices vs. Mikro-Tip^®^ reference probes was determined by Bland–Altman plots of mean vs. differences in ICP. Linear correlation and the coefficient of determination of SmO_2_ values vs. reference probe ICP were determined using the Pearson correlation coefficient. Significance for all analyses was determined using 2-sided test with an alpha of 0.025 unless otherwise stated.

## 3. Results

**Figure 2:**
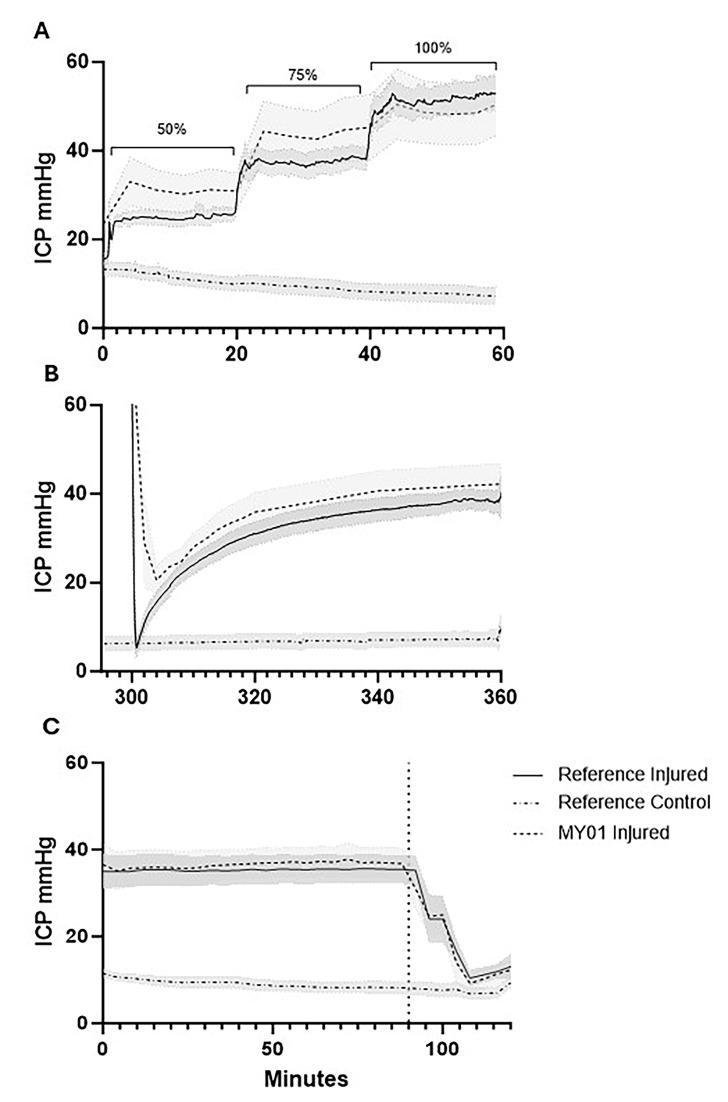
Intracompartmental limb pressure quantified by MY01^®^ vs. reference probes. (**A**) Intracompartmental pressure graphs during stepwise balloon inflation (50%, 75%, and 100% of diastolic blood pressures, indicated by brackets); (**B**) reperfusion following balloon deflation; (**C**) 24 h post-injury pre- and post-fasciotomy (indicated by vertical dotted line). Intracompartmental pressures during ischemia exceeded the upper limit of detection of the MY01^®^ device. Shaded regions indicate SEM of limb pressures (*n* = 8). ICP, intracompartmental pressure.

During incremental intracompartmental balloon inflation (pre-ischemia phase), ICP reference probe values were maintained near the targets of 50, 75, and 100% of diastolic pressure, with values of 48.86 ± 6.11%, 72.63 ± 5.34%, and 98.08 ± 6.73%, respectively. The control limbs showed an ICP that was 19.59 ± 11.50% of diastolic pressure during this period (Figure 2A). During the ischemia phase, ICP was maintained at a pressure of 30 mmHg greater than MAP over a four-hour period. During this time, the ICP exceeded the upper limit of detection (99 mmHg) of the MY01^®^ device. Therefore, it was not possible to collect sufficient data with the MY01^®^ device during this phase. Subsequent deflation of the balloon angiocatheter produced an immediate reduction in the injured limb reference probe ICP (6.34 ± 6.17 mmHg) comparable to the control limb ICP (6.38 ± 4.59 mmHg). However, during the following 60 min, a spontaneous increase in ICP was observed within the injured limb, consistent with reperfusion injury. At the conclusion of this reperfusion phase, the mean reference probe ICP was 38.71 ± 6.03 mmHg within the injured limb and 7.14 ± 4.88 mmHg within the control limb (Figure 2B). Twenty-four hours after injury onset, the injured limbs demonstrated a similar ICP of 35.58 ± 8.75 mmHg vs. 9.05 ± 3.18 mmHg within control limbs (Figure 2C). Subsequent fasciotomy within the injured limbs resulted in an immediate reduction in ICP to levels similar to those observed in the control limb.

**Figure 3:**
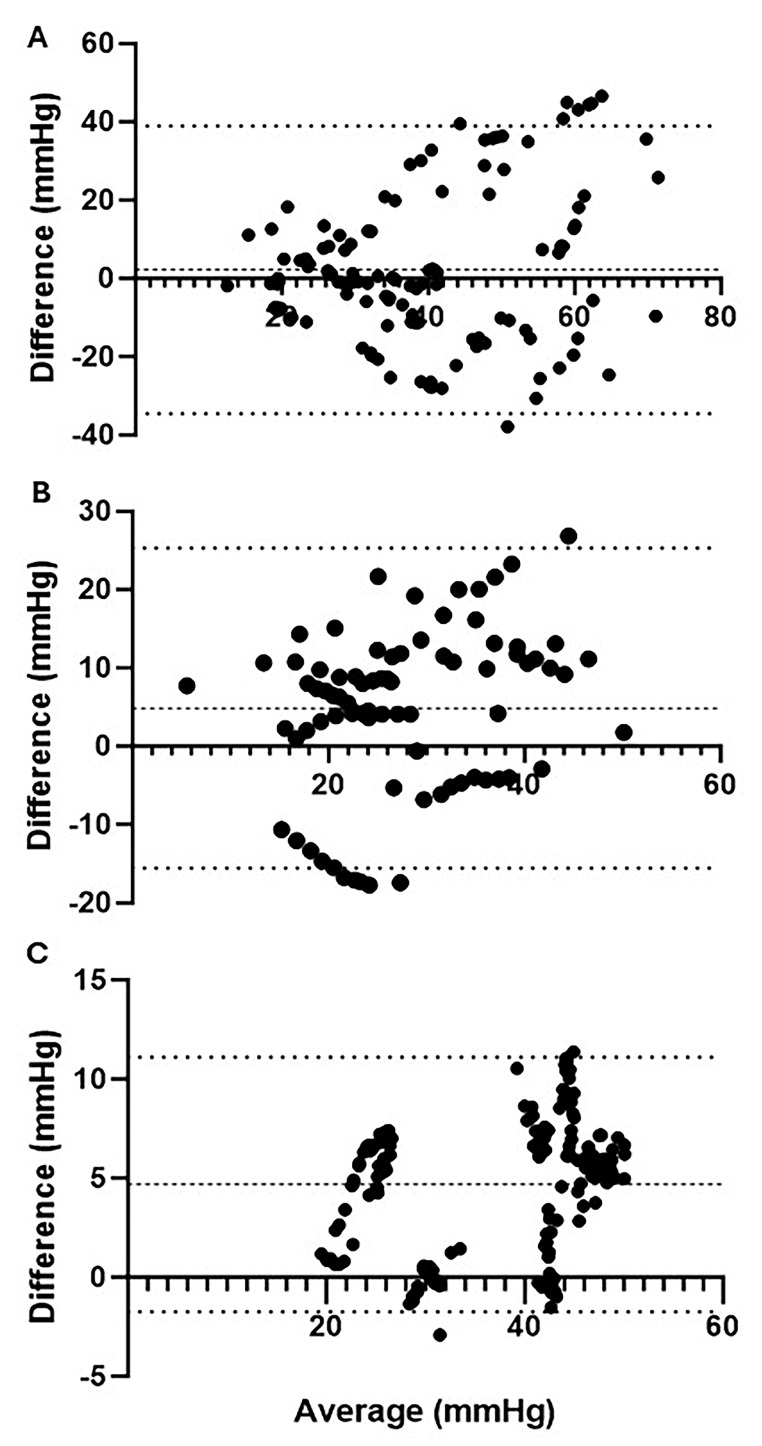
Bland−Altman plots of ICP quantified by reference probes vs. MY01^®^. Mean ICP vs. differences in ICP quantified by reference probes and MY01^®^; (**A**) During pre-ischemia, there was a mean bias of 2.21 ± 18.77 (95% CI: −34.58–39.00) mmHg compared to reference probes; (**B**) during reperfusion, there was a mean bias of 4.86 ± 10.43 (95% CI: −15.57–25.30) mmHg compared to reference probes; (**C**) 24 h post-injury there was a mean bias of 4.69 ± 3.28 (95% CI: −1.74–11.11) mmHg compared to reference probes.

Commercially available devices measuring ICP or muscle oxygenation were utilized across each phase of injury for comparison against ICP reference probes (Table 1). During the pre-ischemia phase, the reference ICP probes and MY01^®^ device demonstrated a mean difference (bias) of 2.21 ± 18.77 (95% CI: −34.58–39.00) mmHg (Figure 3A). During the ischemia phase, where ICP was maintained at levels more than 30 mmHg above MAP, ICPs exceeded the upper linear limit of 99 mmHg for the MY01^®^ device. Consequently, correlative data between the reference probes and MY01^®^ device cannot be reported for this phase. During the reperfusion phase, the mean difference between ICP reference probes and MY01^®^ was 4.86 ± 10.43 (95% CI: −15.57–25.30) mmHg (Figure 3B). Finally, at 24 h post-injury, the mean difference in reference probe ICP and MY01^®^ was 4.69 ± 3.28 (95% CI: −1.74–11.11) (Figure 3C). The MY01^®^ demonstrates a strong positive correlation with paired mean reference probe values at 24 h post-injury (r = 0.86, R^2^ = 0.73, *p* < 0.0001), although during pre-ischemia (r = 0.39, R^2^ = 0.15, *p* < 0.0001) and reperfusion (r = 0.52, R^2^ = 0.27, *p* < 0.0001), a higher degree of variability was observed.

**Figure 4:**
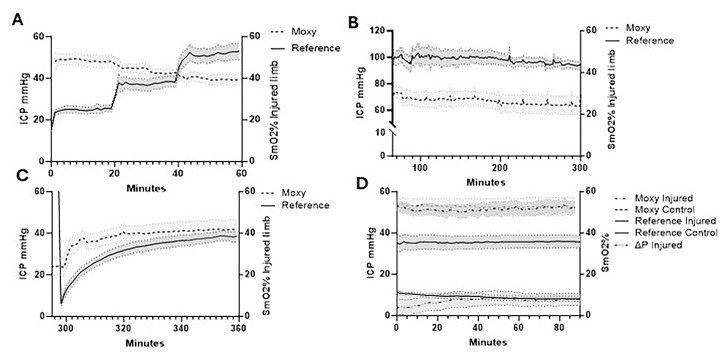
Intracompartmental pressure changes vs. percent SmO_2_. (**A**) Intracompartmental pressure changes vs. percent SmO_2_ during stepwise balloon inflation (50%, 75%, and 100% of diastolic blood pressures); (**B**) prolonged ischemia; (**C**) reperfusion following balloon deflation; (**D**) 24 h post-injury pre-fasciotomy. Injured limb intracompartmental and perfusion pressure and control limb intracompartmental pressure are shown. Shaded regions indicate SEM of limb pressures (*n* = 8). ICP, intracompartmental pressure.

In addition to direct ICP measurements, the Moxy Monitor was used to measure muscle SmO_2_ (Figure 4). SmO_2_ measurements during the stepwise pre-ischemia phase were 46.01 ± 11.20%, 44.81 ± 6.06%, and 40.68 ± 6.11% relative to ICP at 50, 75 and 100% of diastolic pressure. During the same period, control limbs had a mean SmO_2_ of 47.83 ± 10.03% (Figure 4A). During the prolonged ischemia period, SmO_2_ rapidly declined to 26.67 ± 9.92% in injured limbs, while control limbs maintained a similar SmO_2_ of 51.04 ± 8.28% (Figure 4B). After balloon deflation, SmO_2_ in the injured limb increased to 41.84 ± 12.12% over the one-hour reperfusion period, while the control limb remained steady at 55.43 ± 7.55% SmO_2_ (Figure 4C).

The Pearson correlation coefficient between mean ICP and SmO_2_ comparing paired values showed a weak negative correlation (r = −0.32, R^2^ = 0.10, *p* < 0.001) during pre-ischemia. Assessing the correlation between paired reference probe ICP and SmO_2_ values in the reperfusion period demonstrated a weak positive correlation (r = 0.10, R^2^ = 0.01, *p* < 0.05). At 24 h post-ischemia, the injured limb exhibited only a minimal reduction in SmO_2_ (1.30 ± 0.53%, *p <* 0.05) relative to the uninjured limb, despite a perfusion pressure (∆P) well below accepted thresholds for ACS (7.20 ± 9.19 mmHg) and a substantial mean difference in ICP between the injured and control limbs (26.53 ± 9.84 mmHg) (Figure 4D).

**Figure 5:**
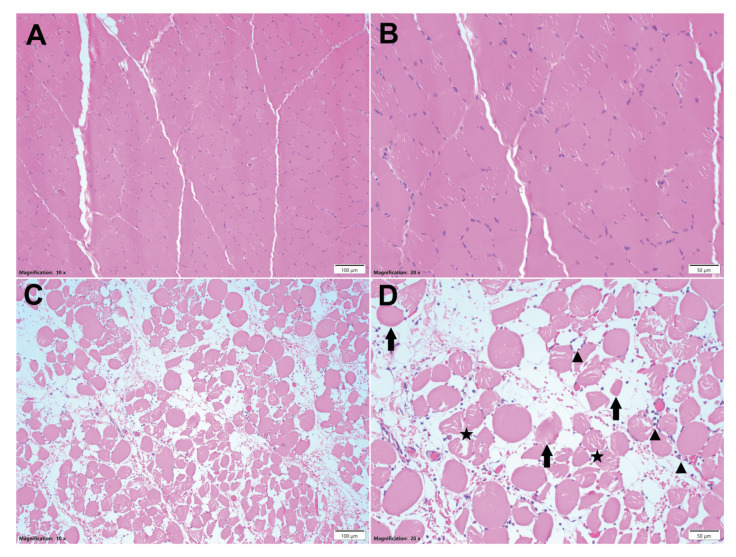
Histologic images of control and injured muscle. Representative H & E staining of full cross sections of peroneus tertius muscles. (**A**) Muscle example of control limb, 10× magnified, scale bar = 120 µm; (**B**) muscle example of control limb, 20× magnified, scale bar = 500 µm. Muscle of control limb showed no histologic abnormalities; (**C**) muscle example of injured limb, 10× magnified, scale bar = 100 µm; (**D**) muscle example of injured limb, 20× magnified, scale bar = 50 µm. Within the injured limbs, myocytes exhibit polyphasic degenerative and necrotic changes characterized by pale, swollen, and vacuolated sarcoplasm (star) with disrupted myofibrils (degeneration); hypereosinophilic, shrunken, and fragmented or hyalinized sarcoplasm (arrows) with loss of cross striations and a pyknotic or karyorrhectic nucleus (necrosis); or rarely have lightly basophilic sarcoplasm with multiple internalized, linearly arranged, vesiculate nuclei with prominent nucleoli (regeneration). Rarely, multifocal random foci of inflammatory cells (▲) expand the epimysium, perimysium, and endomysium, and surrounded and separate individual myocytes, composed primarily of neutrophils and eosinophils.

Histological analysis of injured and uninjured muscle tissue was performed 24 h after injury. This revealed significant pathological changes in the injured muscle compared to uninjured muscle (Figure 5). Between 50% and 95% of the cross-sectional area of the injured muscle showed signs of damage, degeneration, necrosis, and atrophy (Supplementary Material S2: Histologic Evaluation). Acute hemorrhages and moderate to marked edema were observed within the muscle, with 50 to 80% of the specimen area affected by edema.

## 4. Discussion

This study evaluated two commercially available devices in a swine model of ACS using an intracompartmental balloon technique. The MY01^®^ device demonstrated a strong correlation with reference ICP probes at 24 h post-injury, while maintaining a bias of less than 5 mmHg across all experimental phases below the threshold of ischemia. These results support its potential as a reliable portable alternative to high-fidelity-pressure catheters. Conversely, the Moxy Monitor showed limited diagnostic utility, with no correlation between SmO_2_ values and ICP along with normal SmO_2_ readings at 24 h, despite perfusion pressures well below established ACS thresholds.

The MY01^®^ has been successfully utilized by the manufacturer in a preclinical swine model by Honjol et al. and several clinical case reports and series [13,25,26,27,28]. Our study expands on these findings providing independent validation of the MY01^®^ as a reliable alternative to continuous ICP monitoring with high-fidelity catheters, emphasizing its potential as a powerful diagnostic tool given its portability and ease of use in the clinical setting. Unlike traditional slit-catheter systems, the MY01^®^ employs electromechanical sensing with continuous monitoring capabilities, representing a significant advancement over single time-point measurement devices and those that rely on a fluid column, both of which are still prevalent in clinical practice [23]. In addition, the objective, continuous ICP measurement can be supported by other trained medical personnel, which reduces the burden on the physician.

Beyond device evaluation, this study incorporated refinements to an ACS model validated in prior work [24,25,29]. Previous studies have demonstrated that volumetric displacement via an intracompartmental balloon compromises muscle perfusion and induces diffuse injury by rapidly raising the ICP [19,24,29,30,31], elevating it beyond a critical threshold, triggering a rapid SmO_2_ decline [19]. Additionally, sustained ischemia followed by reperfusion has been shown to produce a spontaneous rise in ICP [29]. Our study builds upon this methodology by incorporating stepwise reductions in perfusion pressure prior to ischemia, allowing the successful targeting if desired ICP and better simulating the pathophysiology of ACS. Despite a shorter ischemic and reperfusion window compared to prior literature, our model reliably demonstrated sustained ICP elevations post-reperfusion, with values exceeding 30 mmHg in the absence of fasciotomy at 24 h [32]. Furthermore, by extending the evaluation to 24 h post-injury, we captured a phase of injury progression seldom explored in prior studies [25]. During this phase of injury, ICP throughout the compartment showed higher uniformity with supporting histologic data showing a preponderance of edema. The shorter ischemic duration may also help minimize muscle damage from balloon inflation itself, allowing for more precise quantification of serum biomarkers and necrosis as a direct consequence of ACS in future work.

A key observation related to the use of a balloon model was the variability in ICP measurements between duplicate reference probes and the MY01^®^ device, which corresponded to the mechanism of ICP elevation. Variability was most pronounced when ICP was elevated via volumetric displacement, likely due to nonuniform force (pressure) distribution from the balloon. Given its cylindrical shape, muscle displacement is greatest at its midline and diminishes peripherally. This variability largely resolved when ICP elevations were induced physiologically, such as during reperfusion and at 24 h post-ischemia. However, even during reperfusion, dynamic ICP fluctuations and related regional pressure variations associated with the probes’ spatial arrangement contributed to additional variance compared to 24 h post-injury.

Our study incorporated commercially available devices selected with an emphasis upon portability, ease-of-use, and diverse modalities. This included conventional ICP quantitation and surrogate metrics of perfusion. While each device supported continuous measurement, thereby limiting error introduced by sampling data at dispersed time points, considering each device’s outcomes throughout the individual phases of the experimental model provides the greatest insight into their performance. Preclinical studies demonstrated an inverse correlation between ICP and SmO_2_ during ischemia [19,23]. Translation to the clinical setting reproduced this relationship during ischemia while the diagnostically critical preischemic period showed relative increases and decreases in SmO2 corresponding with hyperemia and the loss of perfusion [16,17,20]. The translation to broader clinical use additionally reported logistical challenges in collecting reliable continuous NIRS data [33]. Despite prior studies suggesting the promise of NIRS for detecting ACS-related perfusion changes, our findings did not support its effectiveness as a non-invasive diagnostic adjunct. Although the device was responsive to ICP changes during the ischemia phase and SmO_2_ improved towards baseline during reperfusion, surprisingly, SmO_2_ values measured 24 h post-ischemia showed a comparatively small mean difference (1.30 ± 0.53%) between injured and control limbs despite the mean ICP differences in limbs being 26.53 ± 9.84 mmHg and the perfusion pressure being 7.20 ± 9.19 mmHg.

This study is not without limitations. Limiting post-reperfusion time points to the initial 60 min and 24 h post-ischemia potentially excluded portions of the physiologic continuum of events that may have further characterized injury progression and better informed the utility of the tested diagnostic methods. Selecting lateral recumbency as a position during data collection may have introduced a minor degree of variance between limbs during data collection. This position was selected in lieu of a supine or sternal position whose effect upon perfusion pressure or ICP, respectively, would have confounded collected data to a much greater degree. This study was conducted utilizing eight female Yorkshire swine; future studies will seek to include a more comprehensive selection of animals. While ACS of the extremity represents an end-state outcome precipitated by a diverse range of injuries, any experimental preclinical model is intrinsically limited to a subset of injury procedures at the exclusion of others which may limit the generalizability. Nonetheless, we believe our model sufficiently replicates key clinical features to support the diagnostic utility of the tested devices. Considering device limitations, the MY01^®^ is unable to detect limb pressures above 99 mmHg. We do not feel the intended clinical application of the MY01^®^ would be impacted by this limitation as continuous ICP monitoring seeks to identify limb pressures approaching ischemia, and a value > 99 mmHg represents a stage of injury progression where surgical intervention is warranted. Additionally, when interpreting the results of the NIRS device, it is important to consider potential confounders that may have influenced the measurement, such as tissue edema, probe placement, and skin perfusion.

## 5. Conclusions

In summary, the MY01^®^ device provided accurate ICP measurements comparable to the reference standard, supporting its potential role in clinical ACS monitoring. In contrast, NIRS as measured by the Moxy Monitor, failed to demonstrate reliable correlations between SmO_2_ and ICP, underscoring potential limitations in its clinical utility and a need for further refinement of non-invasive diagnostic adjuncts for this intended purpose.

## Figures and Tables

**Table 1:** Data table with results of reference probes, MY01^®^ and Moxy Monitor at different study phases.

Parameter	Evaluation	Pre-Ischemia			Ischemia	Post-Ischemia		
		−50% ΔP	−75% ΔP	−100% ΔP	60–300 min	Initial (300 min)	1 h (360 min)	24 h
ICP, Reference probe mean	Injured	48.86 ± 6.11%	72.63 ± 5.34%	98.08 ± 6.73%	97.28 ± 18.30	6.34 ± 6.17	38.71 ± 6.03	35.58 ± 8.75
	Control	19.59 ± 11.50%			5.06 ± 4.43	6.38 ± 4.59	7.14 ± 4.88	9.05 ± 3.18
ICP, Difference between means	Reference probe technical replicates	10.37 ± 10.73			31.61 ± 39.68	7.24 ± 6.92		2.85 ± 1.70
	MY01^®^ vs. reference probes	14.00 ± 12.64, 2.21 CI			above instrument linearity	10.33 ± 11.82, 2.47 CI		5.56 ± 2.67, 0.39 CI
SmO_2_, Moxy	Injured	46.01 ± 11.20	44.81 ± 6.06	40.68 ± 6.11	26.67 ± 9.92	26.67 ± 9.92	41.84 ± 12.12	51.56 ± 8.26
	Control	47.83 ± 10.03			51.04 ± 8.28	51.04 ± 8.28	55.43 ± 7.55	52.86 ± 10.46
SmO_2_ vs. reference ICP Correlation	Grouped	0.9405 R^2^			0.5419 R^2^	0.9279 R^2^		0.2097 R^2^
	Individual	0.2096 R^2^			0.0118 R^2^	0.0108 R^2^		0.0027 R^2^

Values are listed as mean mmHg ± SD unless otherwise indicated. ΔP: perfusion pressure; ICP: intracompartmental pressure; SmO_2_: tissue oxygenation.

## Data Availability

Due to the size of the raw files, datasets are available upon request.

## Supplementary Materials

The following supporting information can be downloaded at https://www.mdpi.com/article/10.3390/diagnostics15202607/s1, Supplementary Material S1: Picture: Experimental Set Up; Supplementary Material S2: Histologic Evaluation.

## Abbreviations

The following abbreviations are used in this manuscript:

ACS	Acute compartment syndrome
COTS	Commercially of the shelf
ICP	Intracompartmental pressure
IM	Intramuscular
IV	Intra vascular
LED	Light-emitting diodes
MAP	Mean arterial pressure
MEMS	Microelectromechanical System
Moxy	Muscle Oxygen (Moxy) Monitor
NIRS	Near-infrared spectroscopy
SD	Standard deviation
SmO_2_	Muscle oxygen saturation
∆P	Perfusion pressure
