# Supplementary material for: Evaluation of Emerging Technologies to Aid in the Detection and Diagnosis of Acute Extremity Compartment Syndrome
Source: Diagnostics (Basel). 2025 Oct 16;15(20):2607. doi: 10.3390/diagnostics15202607 (PMC12564605; doi:10.3390/diagnostics15202607)
Supplement: Supplementary file 1 [file diagnostics-15-02607-s001.zip › Supplementary Material 2.pdf]

Supplementary Material S2: Histologic evaluation

Histologic Evaluation of Injured and Uninjured Muscle at 24 Hours Post Injury

Animal #	Leg	Site (Muscle)		Muscle D/N/L	Predominant Muscle Change	IM Hemorrhage	IM Edema	IM Edema % of slide	Vascular Congestion 0, 1-25%, 2-50%, 3-75%, 4-100%	Vascular Occlusion (thrombi) 0, 1-25%, 2-50%, 3-75%, 4-100%	Overall total inflammation 0 = none, 1 Minimal, 2 mild, 3 Moderate, 4 Severe	Neutrophils (NT)	Eosinophils (EO)	Macrophages (MO)	Lymphocytes/plasma cells (LP)	Fibroblasts (FB)	Comments
1	L	PT		0	0	0	0	0	0	0	0	0	0	0	0	0	
2	R	PT		3	4	1	3	70%	3	1	1	1	1	0	0	0	Approx 75% muscle affected w/ damage
3	L	PT		0	0	0	0	0	0	0	0	0	0	0	0	0	
3	R	PT		4	4	1	4	80%	3	1	1	1	1	0	0	0	Approx 90% muscle affected w/ damage
4	L	PT		0	0	0	0	0	0	0	0	0	0	0	0	0	
4	R	PT		4	4	1	4	80%	3	1	1	1	1	0	0	0	Approx 95% muscle affected w/ damage
5	L	PT		0	0	0	0	0	0	0	0	0	0	0	0	0	
5	R	PT		3	4	1	3	70%	3	1	1	1	1	0	0	0	Approx 75% muscle affected w/ damage
6	L	PT		0	0	0	0	0	0	0	0	0	0	0	0	0	
6	R	PT		3	4	1	4	65%	3	1	1	1	1	0	0	0	Approx 75% muscle affected w/ damage
7	L	PT		0	0	0	0	0	0	0	0	0	0	0	0	0	
7	R	PT		4	4	1	3	80%	3	1	1	1	1	0	0	0	Approx 95% muscle affected w/ damage
8	L	PT		0	0	0	0	0	0	0	0	0	0	0	0	0	
8	R	PT		3	4	1	3	70%	2	1	1	1	1	0	0	0	Approx 75% muscle affected w/ damage
9	L	PT		0	0	0	0	0	0	0	0	0	0	0	0	0	
9	R	PT		2	4	1	2	50%	1	1	1	1	1	0	0	0	Approx 50% muscle affected with damage

Skeletal muscle changes (degeneration, necrosis, atrophy, loss, regeneration)
0 - No muscle changes noted
1 - ≤25% (% of total skeletal muscle on slide)
2 - 26-50% (% of total skeletal muscle on slide)
3 - 51-75% (% of total skeletal muscle on slide)
4 - 76-100% (% of total skeletal muscle on slide)

Predominant muscle change
0 - No change
1 - Myocyte degeneration and necrosis
2 - Myocyte regeneration (mild)
3 - Myocyte regeneration and satellite hypertrophy (w/ atrophy and loss)
4 - Myocyte degeneration, necrosis, loss, atrophy (w/ low #s of regenerative myocytes)
5 - Myocyte degeneration, necrosis, loss, atrophy, regeneration

Intermyocyte hemorrhage
0 - Absent
1 - Acute hemorrhage only
2 - Acute and chronic hemorrhage
3 - Chronic hemorhage

Intermyocyte edema
0 - None
1 - Minimal
2 - Mild
3 - Moderate
4 - Marked
5 - Severe

Inflammation (neutrophils, histiocytes and lymphocytes/plasma cells)
0 - None
1 - Minimal
2 - Mild
3 - Moderate
4 - Marked
5 - Severe
