# Supplementary figures and images for: Evaluation of Emerging Technologies to Aid in the Detection and Diagnosis of Acute Extremity Compartment Syndrome
Source: Diagnostics (Basel). 2025 Oct 16;15(20):2607. doi: 10.3390/diagnostics15202607 (PMC12564605; doi:10.3390/diagnostics15202607)

**Supplementary Material S1: Picture: Experimental Set Up**

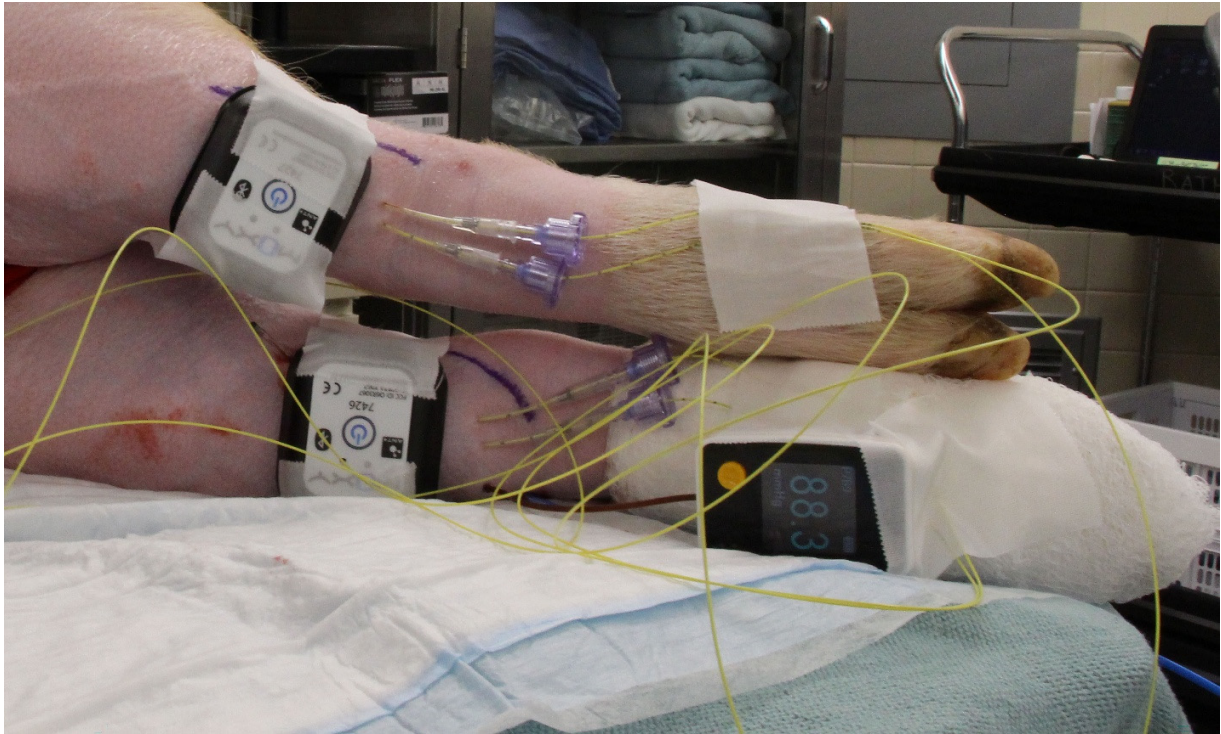

Supplement: Supplementary file 1 [file diagnostics-15-02607-s001.zip › Supplementary Material 1.pdf]
